# A Giant Case of Pyonephrosis Resulting from Nephrolithiasis

**DOI:** 10.1155/2014/161640

**Published:** 2014-07-03

**Authors:** Ali Erol, Soner Çoban, Ali Tekin

**Affiliations:** ^1^Department of Urology, Medical Park Hospital, Istanbul, Turkey; ^2^Department of Urology, Sevket Yilmaz Education and Research Hospital, Yildirim, Bursa, Turkey; ^3^Department of Urology, Duzce University School of Medicine, Duzce, Turkey

## Abstract

Pyonephrosis is an uncommon disease that is associated with suppurative destruction of the renal parenchyma in adults. Upper urinary tract infection and obstruction play a role in its etiology. Immunosuppression from medications (steroids), diseases (diabetes mellitus, AIDS), and anatomic variations (pelvic kidney, horseshoe kidney) may also be risk factors for pyonephrosis. Fever, shivering, and flank pain are frequent clinical symptoms. On physical examination, a palpable abdominal mass may be associated with the hydronephrotic kidney. Septic shock and death can occur if the disorder is not treated with urgent surgery. After the acute phase, most patients are treated with nephrectomy. In this paper, we share the etiology, clinical features, diagnosis and treatment of pyonephrosis using the background of a case with giant pyonephrosis developing due to a kidney stone, the most common cause of upper urinary tract obstruction.

## 1. Introduction

Pyonephrosis is a disease causing suppurative destruction of the renal parenchyma. If it is not diagnosed early, it can worsen rapidly and cause the death of the patient with the development of septic shock. Clinical findings of the patients vary from asymptomatic bacteriuria (15%) to sepsis. Fever, chills, and flank pain are most commonly seen.

Radiological tests such as ultrasound (USG), computed tomography (CT), urography, and magnetic resonance imaging (MRI) are used in the diagnosis of pyonephrosis. If the pus detected as a result of the investigations is not surgically drained, antibiotics may not be very effective. In this context percutaneous or open nephrostomy or ureteral catheter insertion is appropriate. However, nephrectomy can be considered as a good treatment option if the contralateral kidney is intact in case of a damaged kidney that has lost most of its functions [1, 2].

## 2. Case Presentation

**Figure 1:**
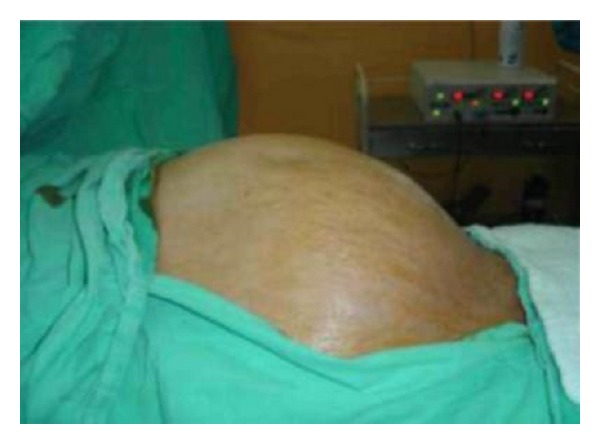
Preoperative view of the abdomen.

Our case was a 65-year-old male who suffers from left flank pain for 4 years and developed abdominal distention 1 month ago. Left hydronephrosis was found by the investigations performed (Figure 1).

**Figure 2:**
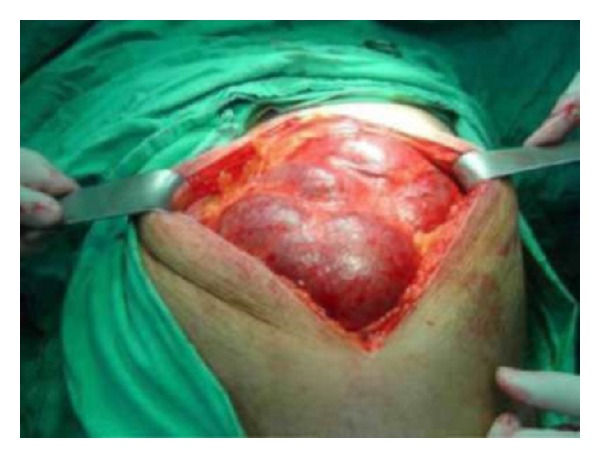
Intraabdominal appearance due to left pyonephrosis during surgery.

Whole abdomen CT revealed a normal right kidney but a cystic mass 13 × 24 × 34 cm in size including calcified areas in the cystic density, without a solid component but with multiple thin septa completely filling the abdomen and passing from the midline to the right in the left kidney region. The patient underwent radical nephrectomy with a diagnosis of nonfunctioning left kidney due to giant pyonephrosis caused by a stone (Figure 2).

**Figure 3:**
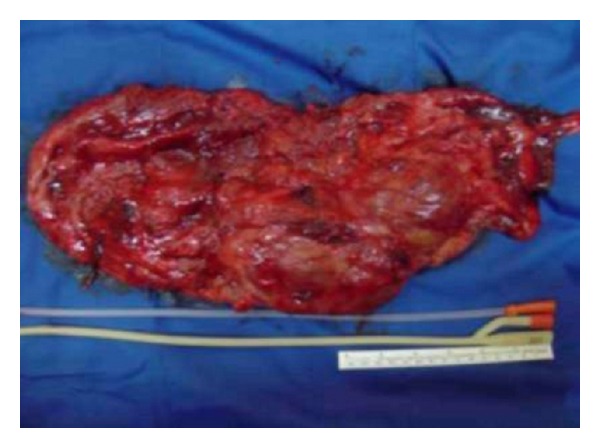
Macroscopic view of the left kidney.

The report from our pathology laboratory was “hydronephrosis and chronic pyelonephritis” (end-stage renal tissue). The macroscopic appearance was nephrectomy material 1150 g in weight, 32 × 15 × 5 cm in size, and pink brown in color, showing cystic enlarged nodularity (Figure 3).

**Figure 4:**
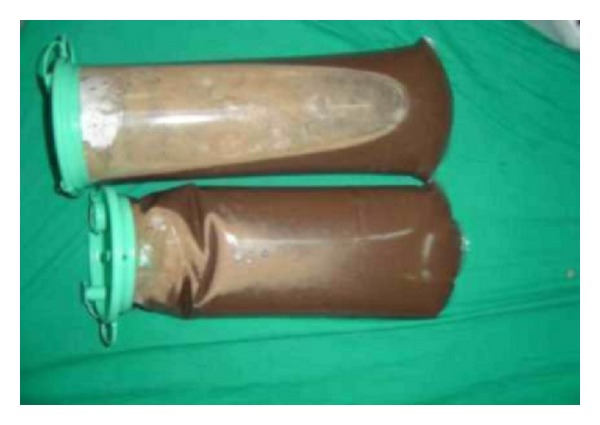
The 7 liters of removed purulent fluid.

Two stones of 1 cm diameter were removed during the operation. Seven liters of purulent material was also drained (Figure 4). No growth was seen in the acid resistant bacillus (ARB), wound site, and mycobacteria cultures taken from the fluid.

## 3. Discussion

Pyonephrosis is a rare disease and upper urinary tract system infections and obstructions play a role in its etiology. There are multiple infectious agents (*Escherichia coli*,* Enterococcus* species,* Candida*,* Klebsiella*,* Proteus*, etc.) in the infection group and stones (staghorn in 75%), yeast balls, metastatic tumors (testicular cancer, colon cancer, etc.), pregnancy, and ureteropelvic junction (UPJ) obstruction in the obstruction group. It may also appear as a complication of past urologic surgery and chronic pyelonephritis.

The accumulation of purulent exudate in the hydronephrotic collecting system and abscess formation constitute the pathophysiology of pyonephrosis [1]. The giant pyonephrosis in our case developed on a background of chronic pyelonephritis due to stone related secondary obstruction, as reported in the literature.

Clinical findings of the patients vary from asymptomatic bacteriuria (15%) to sepsis. Fever, chills, and flank pain are most commonly seen. Rabii et al. found lumbar pain in 70% of 14 pyonephrosis cases, together with painful lumbar region on examination in 5 cases and fever, chills, and pyuria in all cases [2]. The etiology was identified as urinary lithiasis in 71% of the cases [2]. St Lezin et al. found nephrolithiasis in 17 of 23 pyonephrosis cases and performed nephrectomy in 5 cases [3]. Our patient had lumbar pain as the symptom, similar to the literature, and the etiology could be a stone. Pyuria is seen very commonly in pyonephrosis and may sometimes be nonspecific. Bacteriuria, fever, pain, and leukocytosis may be absent in 30% of the cases. No growth was seen in any of the cultures in our case.

USG and CT are the methods generally used for the diagnosis of pyonephrosis. However, CT is more effective than USG as it identifies renal function, causes of obstruction (stone, retroperitoneal fibrosis, metastatic masses, etc.), and abdominal pathologies such as hydronephrosis better. Fultz et al. found that CT was a very sensitive radiological diagnostic method in the study they conducted on 17 pyonephrosis plus hydronephrosis cases [4]. We also obtained diagnostic information with CT in our case.

Antibiotics have no effect in pyonephrosis unless the pus is surgically drained. Percutaneous nephrostomy and urethral catheter insertion are therefore necessary. Studies show percutaneous drainage to be a fast, trusted, and effective diagnostic and therapeutic method [1, 3, 5].

Radical nephrectomy can be the preferred treatment for a kidney that has lost most of its function if the contralateral kidney is normal. Nephrectomy has been found to have fewer complications compared to other treatments [6, 7]. Left primary nephrectomy was performed as the treatment in our case. The patient had no postoperative complications.

We did not find any other case with pyonephrosis of a similar size in the literature. Giant pyonephrosis is rare now due to the advanced diagnostic methods and medical and surgical treatment.

In conclusion, pyonephrosis should be diagnosed early and surgery should be performed immediately. Radical nephrectomy can still be considered as a potential treatment that is curative. However, the best treatment is to detect and treat the stones that play a major role in the etiology.
